# Exploring challenges and opportunities in detecting emerging drug trends: A socio-technical analysis of the Canadian context

**DOI:** 10.17269/s41997-023-00842-w

**Published:** 2023-12-29

**Authors:** Etran Bouchouar, Marissa J. Levine, Samuel Ileka-Priouzeau, Sailly Dave, Allan Fu, Jason L. Salemi

**Affiliations:** 1 College of Public Health, University of South Florida, Tampa, FL USA; 2 Public Health Agency of Canada, Ottawa, ON Canada; 3 Business Technology, Shopify Inc, Ottawa, ON Canada

**Keywords:** Early warning, Substance use, Systems thinking, Surveillance, Alerte précoce, consommation de substances, théorie des systèmes, surveillance

## Abstract

**Objectives:**

This study aimed to apply a systems thinking approach to explore factors influencing the detection of emerging drug trends in Canada’s provinces and territories to better understand how the local context can influence the design and performance of a pan-Canadian (i.e., national) substance use early warning system (EWS). This study also presents a set of actionable recommendations arising from the results.

**Methodology and methods:**

Semi-structured interviews were conducted with 13 purposively recruited Medical Officers of Health and epidemiologists from across Canada working in the field of substance use. Thematic and social network analysis guided by the socio-technical systems framework were subsequently employed.

**Results:**

Barriers and facilitators for detecting emerging drug trends in provinces and territories are a product of the collective linkages and interactions between social (objectives, people, culture), technical (tools, practices, infrastructure), and external environmental (financial, regulatory frameworks, stakeholders) factors. Shortcomings in several of these areas shaped the system’s behaviour and together contributed to fragmented operations that lacked strategic focus, poorly designed cross-sector partnerships, and unactionable information outputs. Participants’ experiences shaped perceptions of a national substance use EWS, with some voicing potential opportunities and others expressing doubts about its effectiveness.

**Conclusion:**

This study highlights interconnected social, technical, and external environmental considerations for the design and implementation of a national substance use EWS in Canada. It also demonstrates the value of using the socio-technical systems framework to understand a complex public health surveillance issue and how it can be used to inform a path forward.

**Supplementary Information:**

The online version contains supplementary material available at 10.17269/s41997-023-00842-w.

## Résumé

**Objectifs:**

Cette étude visait à appliquer une approche de pensée systémique pour explorer les facteurs influençant la détection des tendances émergentes en matière de drogues dans les provinces et territoires (P/T) du Canada afin de mieux comprendre comment le contexte local peut influencer la conception et le rendement d’un système pancanadien (c.-à-d. national) d’alerte précoce (SAP) en matière de consommation de substances. Cette étude présente également un ensemble de recommandations réalisables découlant des résultats.

**Méthodologie et méthodes:**

Des entrevues semi-structurées ont été menées auprès de 13 médecins hygiénistes et épidémiologistes recrutés dans tout le Canada et travaillant dans le domaine de la toxicomanie. L’analyse des réseaux thématiques et sociaux guidée par le cadre des systèmes socio-techniques (STS) a ensuite été utilisée.

**Résultats:**

Les obstacles et les facilitateurs à la détection des tendances émergentes en matière de drogues dans les provinces et les territoires sont le produit des liens et des interactions collectifs entre les facteurs sociaux (objectifs, personnes, culture), techniques (outils, pratiques, infrastructure) et environnementaux externes (cadres financiers, réglementaires, intervenants). Les lacunes dans plusieurs de ces domaines ont façonné le comportement du système et, ensemble, ont contribué à des opérations fragmentées qui manquaient d’orientation stratégique, à des partenariats intersectoriels mal conçus et à de l’information inexploitable. Les expériences des participants ont façonné les perceptions d’un SAP national sur la consommation de substances, certains exprimant des opportunités potentielles et d’autres exprimant des doutes quant à son efficacité.

**Conclusion:**

Cette étude met en évidence les considérations sociales, techniques et environnementales externes interconnectées pour la conception et la mise en œuvre d’un SAP national sur la consommation de substances au Canada. Il démontre également la valeur de l’utilisation du cadre STS pour comprendre un problème complexe de surveillance de la santé publique et comment il peut être utilisé pour éclairer une voie à suivre.

## Introduction

Canada faces a complex and evolving addiction and overdose crisis fueled by an increasingly toxic drug supply, with the use of opioids, methamphetamine, and new psychoactive substances (NPS) on the rise (Health Canada & U.S. Department of Health and Human Services, 2022; Cornell, 2014). Identifying emerging drug trends quickly and communicating them effectively to stakeholders are crucial for preventing escalation and minimizing harm. However, gaps in Canada’s data monitoring systems, such as incomplete and untimely information, continue to impede the detection of emerging drug trends in their early stage of development (Abdesselam et al., 2018).

Several countries, including the United States, Australia, the United Kingdom, Belgium, and Norway, have established substance use early warning systems (EWS) to detect and respond to early changes in drug supply, usage patterns, and related harms (Bouchouar, 2022). These EWS cover a broad range of substances (illicit and licit) and populations and use a combination of lagged (e.g., drug seizures) and leading-edge data sources (e.g., online marketplace, key informants), with a focus on real-time monitoring of related harms (Bouchouar, 2022). They employ diverse analytic methods to detect aberrations, conduct risk assessments, and disseminate findings through various channels. Many rely on IT platforms, business intelligence tools, and multi-agency and multi-disciplinary coordination (Bouchouar, 2022). While similarities exist, there is no established standard for a best practice model of an EWS.

While The Canadian Community Epidemiology Network on Drug Use (CCENDU) operates as Canada’s sentinel-based surveillance network with early detection and response capabilities, as of its latest publication, it had yet to fully develop into a formal EWS with more complete and defined components (Young et al., 2015). Cognizant of the growing demand for a Canadian EWS (Health Canada, 2019), CCENDU has expressed interest in expanding its capabilities to fill this void (Young et al., 2015), and in parallel, Health Canada, in partnership with CCENDU and federal, provincial, and territorial partners, is actively working towards developing an EWS as a component of its Canadian Drugs Observatory (Health Canada, personal communication, 2020). While existing international EWS can help inform the design of a Canadian national substance use EWS, there is a need to better understand contextual factors within Canada’s provinces and territories (P/Ts) to effectively tailor the design for the Canadian landscape. P/Ts, as key partners in federal surveillance initiatives, play a significant role in contributing data and insights, ensuring a coordinated and comprehensive national system.

**Fig. 1:**
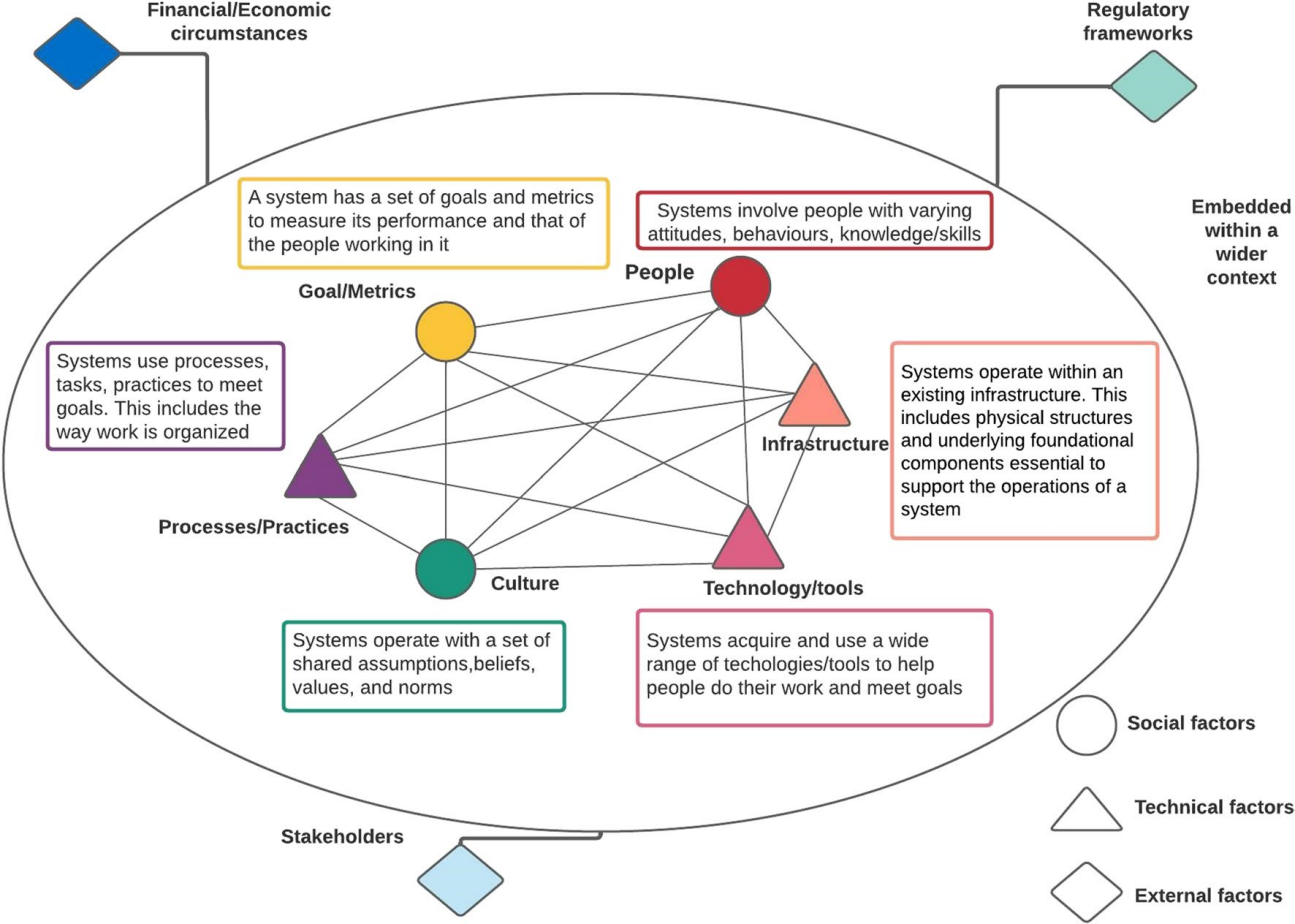
STS framework. Note: Adapted from M.C Davis et al. (2014)

In the domain of public health surveillance, a notable gap exists in the availability of widely applied theoretical frameworks that provide guidance for the design and implementation of surveillance systems. With limited existing use cases, there is a need to explore alternative frameworks that align with the technical and social aspects of public health surveillance and the adaptive nature of health systems (Greenhalgh et al., 2017). In this regard, the socio-technical systems (STS) framework emerges as a promising approach, as it incorporates a systems thinking lens and encompasses relevant social, technical, and external environmental factors (Fig. 1), making it well suited for guiding the exploration of contextual factors that can influence and shape design and implementation of a national EWS (Davis et al., 2014; Hughes et al., 2017). System thinking aids researchers in understanding the interconnectedness among factors that collectively shape a system’s behaviour (Wilkinson et al., 2018). Social network analysis (SNA) also offers valuable methods and measures to enhance understanding of the social factors involved in detecting emerging drug trends within the STS framework, including the identification of influential actors.

Our study aimed to apply STS and SNA to explore the experiences of P/T epidemiologists and Medical Officers of Health (MOHs) in detecting emerging drug trends including barriers, facilitators, and perceptions about a pan-Canadian (i.e., national) EWS. We offer actionable recommendations from the results, which may inform Canada’s substance use EWS and serve as a guide globally.

## Methodology and methods

### Design

We used a pragmatic qualitative descriptive design (Bradshaw et al., 2017) using semi-structured interviews to facilitate in-depth understanding of epidemiologists’ and MOHs’ experiences. This design aligned best with our implementation science–related objective, enabling us to use pragmatic approaches to extend the use of the STS framework (Ramanadhan et al., 2021). Our study, approved by the University of South Florida’s Research Ethics Committee (#002737), ensured participant informed consent, confidentiality, and data security, with records retained in an encrypted laptop securely for 5 years.

### Participants

We purposively recruited epidemiologists and MOHs from across Canada’s P/T public health organizations working in substance use surveillance due to their broad knowledge and lead roles in this area. Epidemiologists were identified via (a) Health Canada’s Data and Evidence Working Group membership list, (b) the Public Health Agency of Canada’s Opioids Public Health Officer’s membership list, and (c) advertising through the Association of Public Health Epidemiologists in Ontario. MOHs were identified by (a) an online search for contact information (e.g., Health Department websites) as well as (b) advertising through Ontario’s MOH local distribution listserv. The eligible participant pool included zero to two epidemiologists and at least one Chief MOH per P/T, along with varying numbers of supporting MOHs depending on population size. All eligible individuals were offered the opportunity to participate, resulting in the recruitment of 13 participants.

### Data generation

Virtual interviews were conducted from August to November 2021 by a single interviewer (EB) and lasted 60–90 min. They were primarily conducted one-on-one, apart from one instance where two epidemiologists from the same jurisdiction were interviewed together at their request. All interviews were audio recorded and transcribed verbatim using Otter.ai, Inc. (2021) (Otter.Ai [software], https://otter.ai/). To ensure accuracy, transcripts were reviewed alongside the audio recording by EB and an external transcriptionist. Questions were aimed at eliciting participants’ knowledge, account of, and experiences with detecting emerging drug trends. A semi-structured interview guide was used containing 11 open-ended and one demographic question (Supplementary material); it was pilot tested by a convenience sample of four epidemiologists to ensure clarity, address any issues, and optimize its effectiveness.

**Fig. 2:**
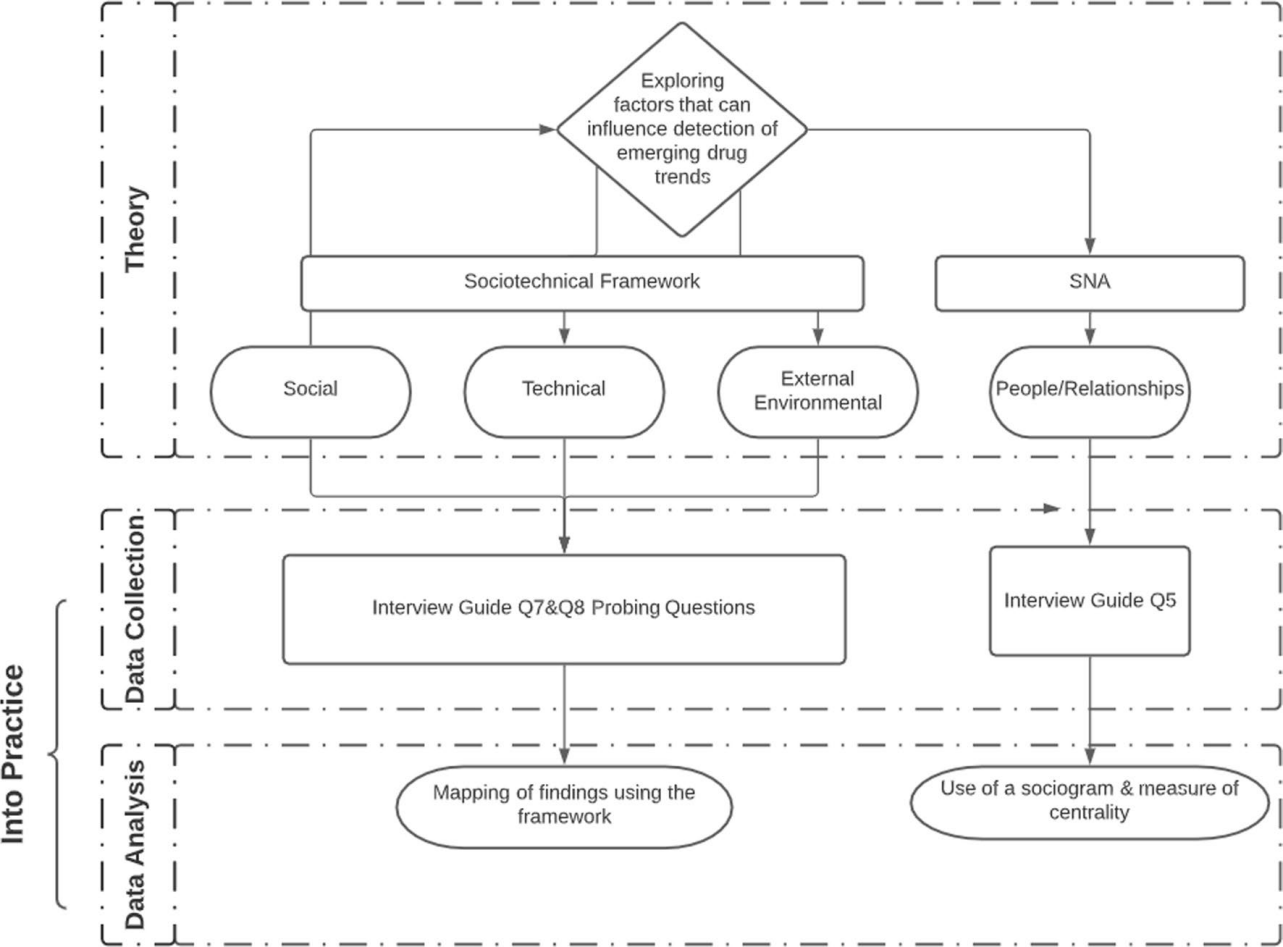
Operationalizing the STS and SNA

To operationalize SNA, participants completed pre-interview question number 5 in the interview guide and responses informed subsequent discussion (Fig. 2). We developed probing questions that drew from the STS framework to elicit information about barriers and facilitators. One question included in the guide focused on statistical detection methods and was only asked to epidemiologists.

### Data analysis

#### Thematic analysis

The data generated underwent analysis incorporating both inductive and deductive strategies. Braun and Clarke’s (2006) six-phase framework guided the inductive thematic analysis process. Transcripts were read and re-read by EB as a whole and line by line actively looking for meaning and patterns in the data. A codebook was developed iteratively and intercoder agreement was assessed on codes developed for two transcripts (one epidemiologist and one MOH) with an epidemiologist who had also pilot tested the interview guide. Discrepancies were discussed and resolved together and applied to previously coded interviews and those that followed. Once all codes were identified, redundancies removed, and preliminary main and subthemes developed, a deductive approach was employed by mapping the codes using the STS framework (Fig. 1). To incorporate multiple perspectives, original coders and one colleague from a diverse professional background reviewed and discussed the themes. EB further reviewed each theme for scope, content, and meaning by re-reading transcripts (Boeije, 2010; Braun & Clarke, 2006). Scientific Software Development GmbH (2021) (ATLAS.ti 9 [software], https://atlasti.com) facilitated data segmentation, annotation, and coding.

We engaged in recruitment, data generation, and analysis simultaneously until we reached data saturation with 13 participants using methods similar to Hennink et al. (2017). Saturation was assessed in code (no new insights and codebook stability) and meaning (comprehending all issues raised). To assess code saturation, we systematically reviewed transcripts, tracking code development in the order they were conducted until the codebook was finalized. We also examined the coding pattern for random order influence. Meaning saturation was evaluated by iteratively reviewing the same codes across interviews, analyzing the various dimensions discussed. By the 5th interview, more than half (53%, *n* = 157) of the codes were identified, and by the 8th interview, 76% (*n* = 224) of new codes were identified, following a similar pattern to the randomized interview order. While additional codes were identified by interview 12, we determined saturation on meaning was reached at this point to fully understand all issues raised and sufficient to answer our research question. Similar studies with focused objectives and homogenous populations reached saturation between the 7th and 12th interviews (Guest et al., 2006; Hennink et al., 2017; Namey et al., 2016; Sebele-Mpofu, 2020). Additionally, our study focused on concrete issues rather than abstract ones, and Hennink et al. (2017) found that concrete codes reached saturation on meaning by interview 9 or earlier, while abstract codes required more interviews.

#### Social network analysis

The characteristics (type of organization, field type, role type) of named key informants were entered into Excel. Responses were reviewed and a relational code name was developed to group and represent the different types of responses captured. Once collated, a frequency analysis was conducted to identify the most common types of organizations and fields of employment held by key informants. A SNA was used to analyze which types of roles were identified most often by participants. Borgatti, S.P., Everett, M.G., and Freeman, L.C. (2002) UCINet 6 for Windows [software] (http://www.analytictech.com/) was used to create a sociogram and to calculate degree centrality to identify prominent influential actors with the highest scores. Similar thematic analysis methods described above were used to understand why key informants were named.

## Results

### Participant characteristics

The 13 participants recruited were from nine (69%) of the 13 jurisdictions in Canada. Most MOHs (three of five) had greater than 5 years of experience in the substance use arena, and the majority (seven of eight) had 1–5 years (Table 1). Despite our attempts, recruitment in Nunavut, Quebec, Nova Scotia, and Newfoundland was not possible due to staffing and time constraints among eligible participants.

**Table 1:** Participant characteristics

Characteristics	Epidemiologist (*n* = 8)	MOH (*n* = 5)	Total (*N* = 13)
Gender			
Male	0	5	5
Female	8	0	8
Years of experience in substance use			
< 1 year	0	1	1
1–5 years	7	1	8
6–10 years	0	2	2
> 10 years	1	1	2
Region in Canada			
The West Coast (BC)	1	1	2
The Prairie Provinces (MB, SK, AB)	2	2	4
The Northern Territories (YT, NWT)	1	2	3
Central Canada (ON)	1	0	1
Atlantic region (PEI, NB)	3	0	3

Participants were from nine of 13 provinces (69% coverage)

*MOH *medical officer of health, *BC* British Columbia, *MB* Manitoba, *SK* Saskatchewan, *AB* Alberta, *YT* Yukon Territory, *NWT* Northwest Territories, *ON *Ontario, *PEI *Prince Edward Island, *NB *New Brunswick

### Thematic and social network analysis

**Fig. 3:**
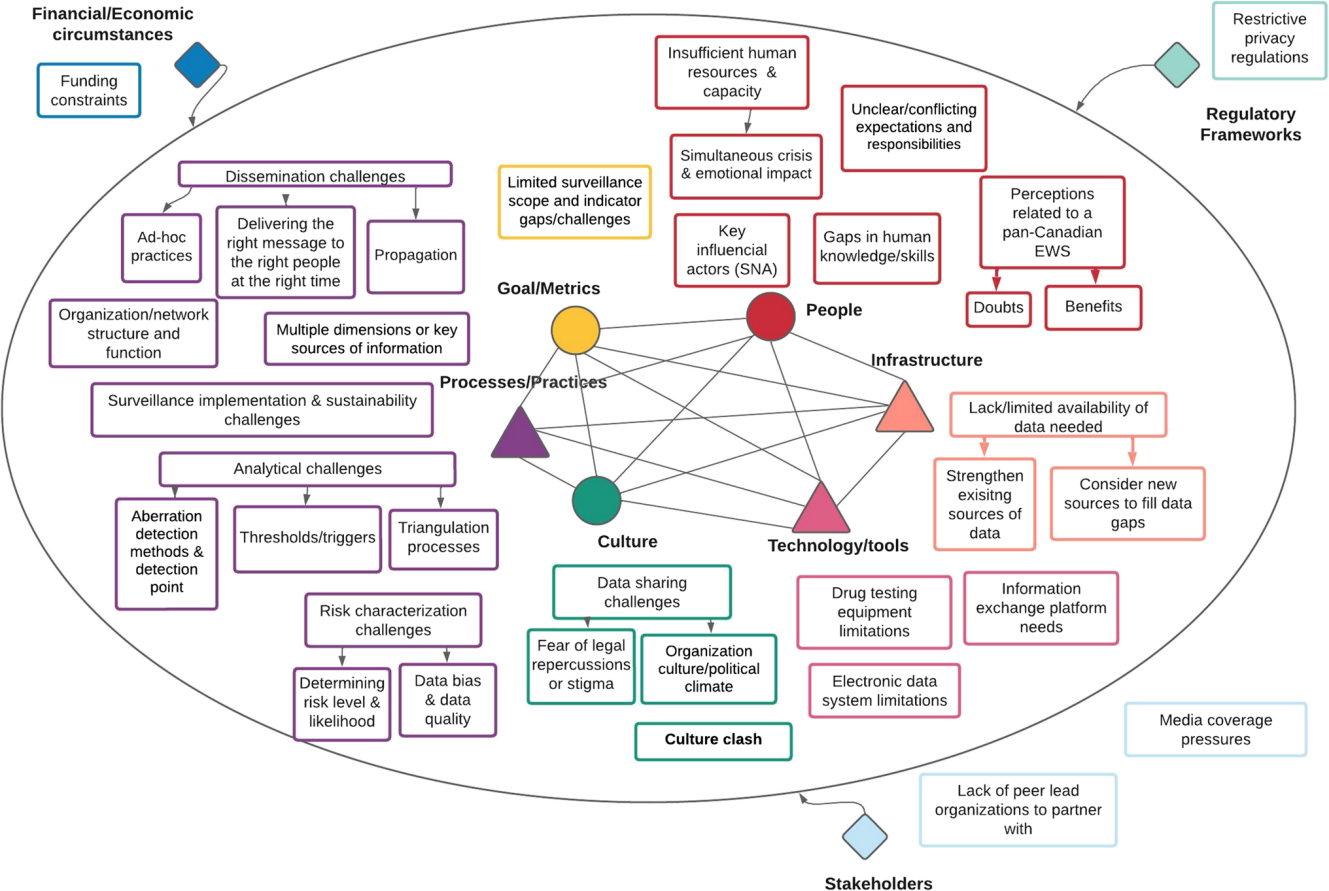
Factors influencing the detection of emerging drug trends in P/Ts from a systems perspective

Key observations from the analysis are mapped onto the nine domains of the STS framework, with the main themes in bold and subthemes italicized (Fig. 3). Along with capturing specific facilitators, participants believed that addressing mentioned barriers would also serve as a facilitator.

#### Social factors—goal and metrics

**Limited surveillance scope and indicator gaps and challenges** were barriers identified by participants. Certain jurisdictions in the Maritimes only focused on opioids while others had a wider scope of substances under surveillance (e.g., methamphetamines). Some participants felt that it was challenging to know what surveillance indicators were most valuable to measure and track. The lack of harm reduction performance measures in Canadian accreditation programs, such as Accreditation Canada for healthcare organizations, was also perceived as a barrier that limited organizations’ commitment to monitoring and improving related outcomes.

P5: So, are we interested in just like, how many drug samples had benzos in them? Or are we interested in what percentage of drugs that were going to primarily be used as an opioid, for example, that someone purchased as fentanyl had benzos in them? So actually, deciding? How do we want to represent the drug supply?

#### Social factors—people

**Fig. 4:**
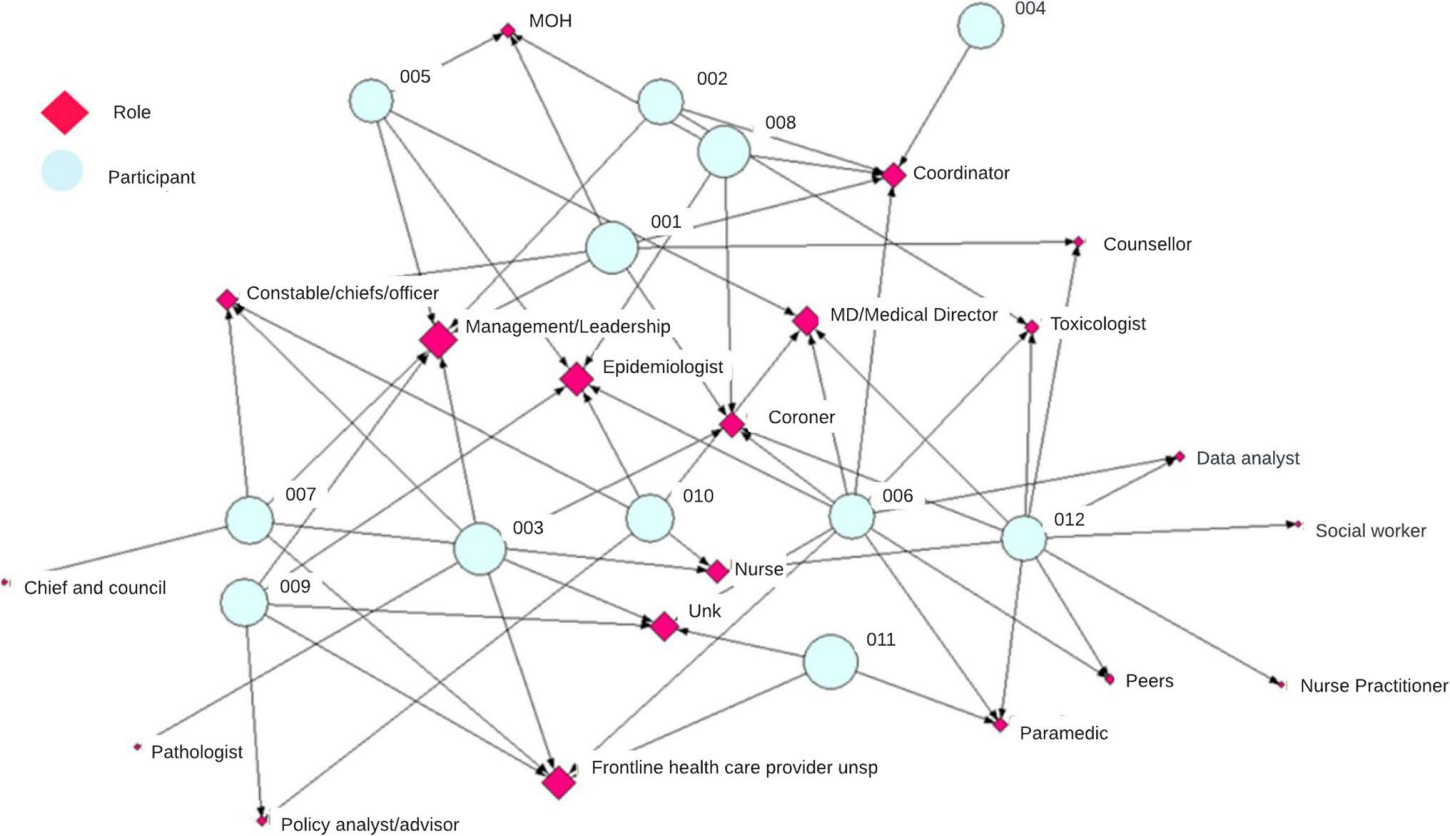
Sociogram of unique types of roles held by key informants (*n* = 19, pink diamond) named by epidemiologists and MOHs (*n* = 12, blue circle) that play a key role in detecting emerging drug trends (size of pink diamond node denotes degree centrality**)**

Several different kinds of professionals were identified as **key influential actors** for the detection of emerging drug trends in P/Ts. On average, each epidemiologist and MOH named seven professionals (range 3–18, median 5) they felt played a key role in identifying emerging drug trends. Most of these professionals were primarily employed in government health departments (26%, *n* = 24/91) and non-government organizations (15%, *n* = 14/91) in the area of mental health/harm reduction (29%, *n* = 26) or public health (11%, *n* = 10). Prominent roles included management/leadership, coordinators (often linked to take-home naloxone programs, needle exchange, and sexually transmitted and blood-borne infection programming), coroners, epidemiologists, and unspecified healthcare providers (Fig. 4). Table 2 provides a summary of their value to participants.

**Table 2:** Key data sources used by participants for detecting emerging drug trends and the reasons for their use

Quantitative sources	
*Acute care*	
EMS	Timely
ED	Geographic information to help target interventions and contextual information surrounding harm (e.g., disposition, naloxone use)
*Law enforcement*	
911 dispatch calls	Geographic granularity to help target interventions
RCMP	Captures community non-fatal ODs and provides insights into the types of locations they are occurring in
*Toxicology and forensics sources*	
Coroner death investigation and toxicology	Detects novel substances with high specificity and reliability; one of the only sources able to detect what substances or combinations are contributing to death; provides emerging changes surrounding context of use
Health Canada Drug Analysis Services	Detects emerging co-occurring substance and novel substances; can delineate which samples are tested for public health purposes that may overcome some inherent biases with the source <https://www.canada.ca/en/health-canada/services/health-concerns/controlled-substances-precursor-chemicals/drug-analysis-service.html>
Drug checking services	Timely; ability to discern whether co-occurring substances were unexpected/expected by end users; ability to detect substances linked to ODs
Urinalysis	Additional population under surveillance (in-treatment); sheds light on consumption patterns; used to triangulate against other tox/forensic sources
*Other *^*a*^	
Pharmacy naloxone	Unknown
Surveys ^b^	Enough of a sample size to characterize populations at risk
Poison control	Coverage on a wide range of substances that may not be captured by other sources
Qualitative—top 5 key informant roles	
Management/leadership	Directly involved with the drug using community and can firsthand see and contextualize emerging trends; they are well connected to professionals and/or initiatives in the harm reduction community and can hear about things secondhand as they are emerging
Coordinators	Ability to contextualize emerging drug trends; ability to detect novel substances before traditional sources
Coroners	Ability to provide detailed contextual information on the circumstances involving the harm that may not be available through other sources
Epidemiologists	Surveillance knowledge and work with multiple data sources; ability to interpret/disseminate findings
Unspecified healthcare providers	Ability to detect early changes in the magnitude of harms; changes in the clinical presentation/harms of PWUDs; contextual information related to use/harms

*EMS* emergency medical services, *ED* emergency department, *RCMP *Royal Canadian Mounted Police, *OD *overdose, *PWUDs *people who use drugs

^a^Identified by a small number of participants

^b^Identified more so by northern and smaller jurisdictions

Along with a diverse range of professionals named, participants discussed **unclear or conflicting expectations and responsibilities** as a barrier. Participants felt roles, responsibilities, and the lead coordinating agent for substance use surveillance were unclear, and connecting with the right people was challenging, which often left them resorting to personal relationships.

P7: I think there’s a lot of back and forth between is this a public health issue? Or is this a mental health and addictions issue? Who’s the lead?

P7: It’s based so much on who you know. So like, I get pulled in the conversation, because somebody who knows me gets pulled into the conversation, there’s not this understanding of who should be involved in the conversations.

At the same time, **insufficient human resources and capacity** (i.e., understaffing and high workload) were widely recognized as barriers to effective surveillance, particularly in rural and northern communities, and were exacerbated by the COVID-19 pandemic. Participants felt that they did not have the bandwidth to manage two *simultaneous crises* and talked about the *emotional impact* of the ongoing substance use crisis and inequitable resource allocation compared to those provided for COVID-19. The need for additional epidemiologists and related supporting staff (i.e., IT to support electronic data systems, data entry staff to improve timeliness, project managers to help bring stakeholders together, and laboratory staff) was voiced.

P1: We’ve just been so swamped with COVID, that, you know, certainly we’ve neglected STBBI harm reduction, drug overdose work, I think there’s no doubt about that.

Participants also described** gaps in human knowledge and skills** in early detection methods, including defining meaningful signals and corroborating them with other data.

P1: What is a signal for a trend? What if you find some cocaine tainted with fentanyl, but you don’t have any other information? …If you have somebody die from a benzo overdose, and maybe there was fentanyl in it, but then you only have one death?

**Perceptions related to a pan-Canadian EWS** were mixed. Participants discussed *benefits*, such as improved situational awareness, ability to anticipate and respond, shared learning, pooled resources and expertise, and enhanced outcomes. A pan-Canadian EWS could also encourage organizations to measure their efforts. However, *doubts* were raised about the system’s ability to gather necessary data due to challenges in data aggregation, access, and coverage. Participants questioned its effectiveness in P/Ts lacking formalized processes or technology to further disseminate findings and in meeting the needs of smaller jurisdictions.

P3: I don’t know what that would look like, how on earth you would get that information to make it an early warning system that is useful to me, as a fantasy will be great.

Overall, participants expressed that the value of the EWS would depend on the design of the system, such as “thinking very carefully about where there can be value add” [P5], ensuring it can connect the dots and “not duplicate some of the ongoing activities” and “tying it to the objective and what decisions and action we’re hoping to influence” [P9].

#### Social factors—culture

Participants identified **data sharing challenges** stemming from a **fear of legal repercussions or stigma** and **organization culture or political climate** as barriers to detection. Some participants also talked about how some First Nation communities may largely be less willing “…to speak about substances in general” and the importance of ownership, control, access, and possession principles (OCAP ®) (https://fnigc.ca/ocap-training/), which ensure First Nations’ data ownership and community benefits, and address historical research issues [P1]. Personal informal relationships facilitated information exchange between stakeholders, but participants found them less ideal due to potential disruptions with staffing changes.

P6: there’s the environment of stigma and discrimination, and people do not sometimes come forward with the knowledge or information that they have, for fear of, you know, punishment for fear of, you know, discrimination against them.

P7: I think the concern is that if we are publishing something, then it is open to public and scrutiny.

Participants identified a **culture clash** between public health and Canada’s acute care–centric health system, which posed a challenge for buy-in towards surveillance systems that do not directly benefit the individual interacting with the system.

P7: The issue with that is it’s not directly helping the patient because by the time we get the test result, the patients usually have been discharged.

Another participant also felt that “health systems aren’t used to paying people who use drugs” [P1], challenging their use as key informants.

#### Technical factors—technology and tools

Nearly all participants talked about **drug testing equipment limitations** as a barrier to detection, advocating for more widespread use of higher discriminatory drug testing equipment in clinical toxicology screening and drug checking services. Some also noted that the type of toxicology panels run in their jurisdiction restricted the detection of new designer drugs.

P5: I think having drug testing that is beyond just like a presence/absence, like fentanyl test strip, I’m talking about, you know, like mass spectrometry, and all the different sub-technologies underneath that.

Participants encountered challenges with **electronic data system limitations** such as some data sources being poorly configured or formatted (e.g., still in paper or functionality to extract from the system in an Excel or csv format is not in place) hindering data access and management. Additionally, participants talked about limitations in current tools for disseminating surveillance-related information (notification, report), with one participant stating “emails just kind of get lost” [P10]. Participants also highlighted **information exchange platform needs, expressing the necessity for an easy** and rapid mechanism to submit and disseminate information. For a pan-Canadian EWS, participants recommended a central portal with customizable dashboards and analytic tools.

#### Technical factors—processes and practices

Participants consider **multiple dimensions and key sources** of information in detecting emerging drug trends, as summarized in Table 2. Laboratory sources including forensic and postmortem played a role in detecting recent trends involving new forms, and increased adulterants, contaminants, and co-occurring substances, with forensic sources detecting changes earlier, and postmortem sources later. Contrastingly, more varied sources, both qualitative (often EMS and ED personnel) and quantitative (Table 2), were discussed for recent trends involving changes in use, harms, or clinical presentation. These detections were perceived to be detected at the peak or late.

**Analytical challenges** were seen as a barrier to the early detection of emerging drug trends. *Aberration detection methods and detection points* used were described by participants, where most relied on descriptive methods (e.g., counts, epi-curves) and their professional judgement to detect aberrations.

P3: most of the data is descriptive, rather than being statistics. And most of it is pretty simple. The line goes up.

The limited use of statistical methods was attributed to small sample sizes, lack of data standardization, limited historical data, and untimely or low time data resolution. Participants also talked about challenges in determining how long the emerging trend was unfolding prior to its first detection, attributing these challenges to “not having enough data points to know” [P2]. Additionally, participants reported having poorly defined *thresholds and triggers*, apart from flags for first-time detections of novel substances. In relation to *triangulation processes*, most participants relied on informal methods such as connecting back with select professionals or reviewing other available sources of data for convergence or divergence. Meaningful thresholds and triggers and an associated decision tool were some components desired in a future pan-Canadian EWS.

Participants also believed **risk characterization challenges** hindered their ability to understand risk and to inform mitigation strategies. Specifically, they voiced difficulty determining *risk level and likelihood* posed by newly detected emerging drug trends. This in part was believed to be attributed to challenges related to (a) rapid access and quick interpretations of toxicology information, and (2) general *data bias and data quality limitations* that make it difficult to characterize risk from a person and place standpoint. Participants expressed the need for a future pan-Canadian EWS to provide the ability to better characterize risk, suggesting (a) establishing a centralized toxicology information resource (e.g., library) drawing from toxicologists, peers, and online forums (e.g., Reddit), (b) modeling the spatial spread of emerging drug trends to better understand introduction patterns across Canada, and (c) establishing high spatial data resolution.

P5: you’d want to have rapid availability of toxicology to interpret, you know, what compound is this? How would you expect it to affect someone who had taken it? Are there any additional measures that people that are using drugs should be aware of that they should take based on the presence of this new compound?

**Dissemination challenges** were also discussed by participants. Many described their practices as ad hoc with no standard processes or decision tools to guide under which circumstances and with whom, and how to communicate emerging drug trends. Several expressed *challenges delivering the right message to the right people at the right time* and avoiding alert fatigue with the ever-changing drug supply. The concept of timely detection was deemed subjective and meaningless if the communicated information lacked concreteness or specificity to impact outcomes.

P5: think also then providing actionable local advice. So, it’s like you so you know, we know the drug supply is toxic. We know that toxicity varies. And, you know, so it’s, so what do you do based on that? You know, this you know, we found that there was it was a little bit more toxic in this one area, this one time, so, what does that actually result in? Apart from sharing the awareness is an important piece. How do you share, for example, pictures of what the drug of concern looks like? That may be something that’s useful that’s sometimes challenging to accomplish.

Participants also described challenges identifying the right target audience and *propagation challenges*, with limited social media use hindering public reach. Slow permissions and approval processes for external sharing and the absence of a public-facing venue for reporting surveillance findings were additional challenges. Regarding **organization and network structure and function**, participants discussed barriers and facilitators. They believed formalized cohesive networks enabled earlier detection, and re-organization in departments facilitated human resource development, capacity building, and information flow improvements. However, the lack of a formalized venue, networks, and associated processes posed barriers.

P7: the struggle is I don’t think we have a formalized system to have these discussions.

Participants also reported **surveillance implementation and sustainability challenges**, highlighting the overlooked sustainability aspect during planning and the resource-intensive yet not always effective implementation of new systems.

P2: the setting up that kind of informal alert system, the network, and then I think it just wasn’t enough oumpth to keep it going. And so it’s, you know, being able to maintain something that you build.

#### Technical factors—infrastructure

The **lack of or limited availability of data needed** was perceived as a barrier; however, participants identified opportunities to *strengthen existing sources of data*, such as (a) timeliness of Drug Analysis Services (DAS) drug seizure forensic lab sample recovery and processing times, (b) implementing real-time or near real EMS and ED harms with syndromic surveillance, and (c) expediting post-mortem tox testing. Participants suggested the need to *consider new sources to fill data gaps* for “hidden populations” who do not interact with health services, such as rural populations, people using “party drugs,” and transient or newly arrived populations. Suggested sources included (a) leveraging crowdsourcing to gather information on unexpected or concerning effects, or hard to gather information such as pictures on drugs of concern, (b) drug checking mail in programs and overdose prevention hotlines, and (c) formal sources for gathering detailed clinical toxicology data in clinical settings. Wastewater was also suggested as a potential source for a future pan-Canadian EWS.

#### External environmental factors—regulatory frameworks

**Restrictive privacy regulations** posed challenges in obtaining and reporting certain data, requiring data sharing agreements. Accessing patient-level clinical and coroner’s data has been particularly challenging for participants.

P5: Because there’s a lot of protocols around how carefully health data are protected. So, data that’s collected in explicitly clinical environment. You know, sometimes there’s barriers to accessing and communicating that data from a patient privacy perspective.

P2: With the, you know, how much Coroners information can we legally obtain? Who can review a record?

#### External environmental factors—financial and economic circumstances

**Funding constraints** have made it difficult to acquire higher discriminatory drug testing equipment and implement clinical toxicology surveillance programs in emergency departments.

#### External environmental factors—stakeholders

External stakeholders mentioned by participants included peer-led organizations and the media. Crowdsourcing-related surveillance systems were seen as difficult to formalize due to distrust and a **lack of peer lead organizations to partner with**. On the other hand, media coverage pressures facilitated access to needed surveillance data. Participants noted that media coverage on substance use increased visibility and pressure on governments to remove existing data sharing barriers to respond to media requests.

## Discussion

Shortcomings in surveillance goals and metrics were identified as a key factor that can shape the function of the broader surveillance system. These limitations are further challenged because the evidence base for substance use early warning indicators and thresholds is limited (i.e., it is not clear which are effective, in what circumstance, and why). The study results indicate that gaps in these areas can contribute to reactive processes that lack strategic focus, resulting in fragmented operations, poorly designed cross-sector partnerships, and unactionable information outputs. Complexities further arise given the multi-sector and multi-disciplinary actors involved in substance use surveillance. Results suggest that failures to clearly define roles and responsibilities prevent joint ownership and alignment of expectations and processes, which can in turn negatively impact detection and response potential. In line with the present study, unclear roles and responsibilities were a major issue in several types of early warning systems (Garcia & Fearnley, 2012; Hosseini et al., 2020; ISDR, 2011; Perera et al., 2019). In our study, these weaknesses may explain why participants depended on informal ad hoc personal relationships and missed opportunities to partner with other key stakeholders outside personal networks. For example, no key informants in the student and youth or nightlife party arena were named, although several substance use EWS depend on them (Mounteney & Leirvåg, 2004; O’Gorman, 2007; Topp et al., 2004).

Similar to the documented experiences of other EWS in the literature, financial constraints and data sharing cultural barriers were key factors influencing investment and access to enabling data sources and technology for earlier detection (ISDR, 2011; Perera et al., 2019; Van Panhuis et al., 2014). Restrictive privacy and drug use legislative frameworks, and the political climate likely contributed to this environment. As such, participants resorted to using several data sources that may not be timely enough or of sufficiently high quality, or harness the right data. The data used are often stored in disparate systems that may not be available in a user-friendly format, further challenging access and data management. Our study results show that these factors may have contributed to highly tedious manual data ingestion and collation processes that trapped human resources, thus limiting capacity to strengthen other parts of the system that are essential to operations. Along with these limitations, weaknesses in analytic capabilities (advanced analysis, triangulation methods, and visualization techniques) prevented organizations from realizing the full potential of their data. Limitations in the data, the lack of well-defined statistical methods used in substance use EWS, and limited technical expertise, human resources, and technology were factors identified as potential contributing factors in this study and others (Mounteney et al., 2011; Perera et al., 2019). All the above factors challenge risk characterization and communication. Additionally, the lack of sophisticated communication systems and related standard operating procedures led to ineffective ad hoc dissemination practices that may not reach the right people at the right time or translate successfully into tactile responses. These results are in line with other studies that found actionable communication to be a major challenge for several kinds of EWS (Budimir et al., 2020; Hosseini et al., 2020; ISDR, 2011; Perera et al., 2019). Overall, these experiences likely shaped the perceptions P/Ts had about a national substance use EWS.

**Fig. 5:**
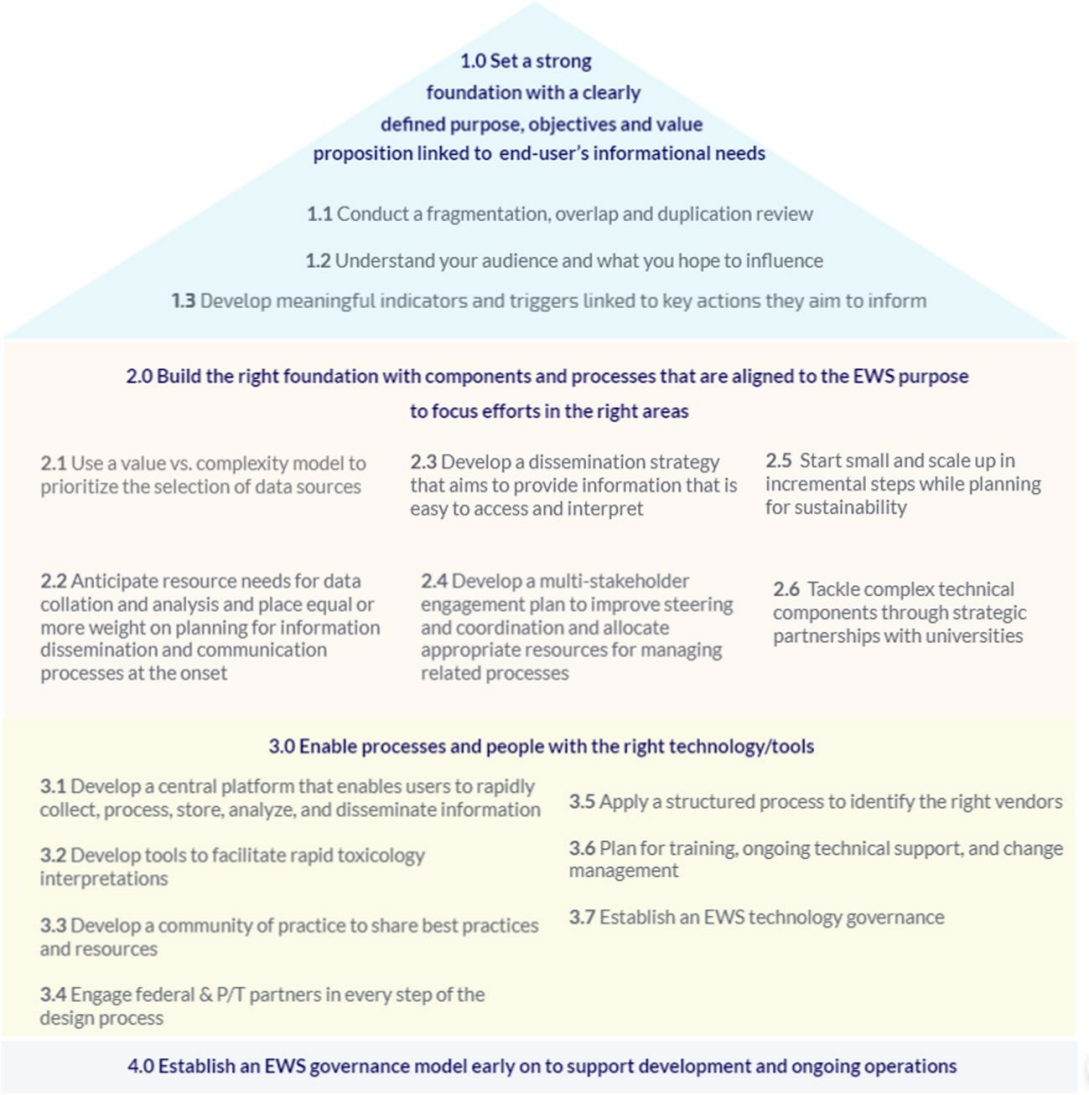
Recommendations for a national substance use EWS in Canada

Results from this study highlight the importance of clear purpose, objectives, and meaningful indicators for aligning processes, technology, and human efforts in the areas that will maximize value, increase efficiency, and minimize waste. These results highlight the role of technology as an enabler of processes and people, not a solution, in complex surveillance initiatives. Additionally, effective dissemination is as critical as data collection and analysis. It is also evident that no single entity will have all the expertise, resources, or time to invest in solving technical methodological issues that can influence detection and risk characterization. Effective partnerships and governance are also key to improving steering and coordination. Recommendations for consideration in the development of a national substance use EWS are presented in Fig. 5.

Our study extends the application of STS to public health surveillance and offers a model for others to build on. STS enabled a holistic exploration, revealing key factors and cross-system relationships influencing early detection, offering a more comprehensive understanding. Ignoring interdependencies may lead to inadequate solutions or unintended effects. Future studies may benefit from incorporating other theories within the STS framework, as we did with SNA to identify influential actors in the people domain.

Our study has some limitations. We could not capture all Canadian jurisdictions’ experiences, and we had more epidemiologist than MOH participants. Despite this, we feel enough participants were recruited to answer our research question. While some results may be transferable to other provinces, we may have missed province-specific experiences. Future research should include all jurisdictions and diverse stakeholders (e.g.., data providers, researchers, and IT specialists) to improve fit and reduce implementation risk.

## Conclusion

To our knowledge, this study is the first to apply a socio-technical systems framework approach to understand contextual factors and their potential influence on a complex public health surveillance initiative. Our results show that our approach can provide a deep understanding of highly intertwined social, technical, and external environmental factors that can be used to inform and improve the design and implementation of a national substance use early warning system in Canada. Our results and the ensuing recommendations may be useful to public health professionals involved in the planning of such a system in Canada. Further, these recommendations and our approach may have great application to other EWS in the public health sphere.

## Contributions to knowledge

What does this study add to existing knowledge?

- Our study builds on the limited literature on contextual factors influencing the detection of emerging drug trends in provinces and territories and the perceptions held by key stakeholders on the development of a national substance use early warning system in Canada.
- It is the first study to apply the socio-technical systems framework, a systems thinking tool, to investigate a complex public health surveillance issue.
- It highlights the significant impact that underlying interconnected social, technical, and external environmental factors have on the performance of surveillance initiatives.

What are the key implications for public health interventions, practice, or policy?

- Our study may assist government officials in Canada to better design a substance use early warning system that is tailored and responsive to the local context.
- Our approach provides a model for public health professionals to use to better understand complex interdependent local contextual factors that can influence positive design and implementation of complex surveillance system initiatives. To date, no such study has been conducted in Canada that provides a framework for use, a first of its kind in Canada.

### Supplementary Information

Below is the link to the electronic supplementary material.

Supplementary file1 (DOCX 19 KB)

## Data Availability

The data that support the findings of this study are available from the first author.
