# Supplementary material for: Exploring challenges and opportunities in detecting emerging drug trends: A socio-technical analysis of the Canadian context
Source: Can J Public Health. 2023 Dec 29;115(2):186–98. doi: 10.17269/s41997-023-00842-w (PMC11006646; doi:10.17269/s41997-023-00842-w)
Supplement: Supplementary file 1 — Supplementary file1 (DOCX 19 KB) [file 41997_2023_842_MOESM1_ESM.docx]

# Semi-structured interview guide

**Introduction**

Emerging drug trends will refer to:

- the introduction of new drugs or new forms of polysubstance use and
- changes in substance use and related harms linked to existing drugs from the way they are used to changes in magnitude or spread into new populations.

**Opening Questions**

1. **What emerging drug trend comes to mind that was recently detected in your jurisdiction?**

Probe:

- At what stage of development was the emerging drug trend when it was detected (i.e., was it at the onset, peak, or at a decline)?

1. **How was this emerging drug trend detected?**

Probe:

- Did you feel this trend was detected in a timely manner?

1. **What could have been done to detect this emerging drug trend sooner?**

**Main Questions**

1. **What sources of information have been key in detecting this or other emerging drug trends in your jurisdiction?**

Probe:

- - Can you name and describe some of these sources?
  - What was particularly useful about them?

1. **Who are some key informants in your jurisdiction, such as those with close links to the drug scene, that play a key role in identifying emerging drug trends in your jurisdiction?** **Please provide the following information for as many people as you feel appropriate.**

| Organization of work | Field of work | Job title | Why they are they considered a key source |
| --- | --- | --- | --- |
|  |  |  |  |
|  |  |  |  |
|  |  |  |  |
|  |  |  |  |
|  |  |  |  |
|  |  |  |  |

1. ***Question of Epidemiologists Only- What are some commonly used statistical signal detection methods that have helped facilitate early detection?**

Probe:

- Is there a preference if multiple are selected and why?
- Are there any protocols for deciding which signals to investigate and which to ignore that you can describe?
- Do you have a process in place you can describe for triangulating signals raised? If yes, please describe this process

1. **What are some challenges or barriers your jurisdiction faces in being able to detect emerging drug trends in their early stage of development?**

Probe:

- - Can you think of any challenges related to human skills/knowledge and resources?
  - Can you think of any challenges related to processes/tasks (e.g., data management, data access, aberration detection methods, data sharing agreements)?
  - Can you think of any challenges related to technology (e.g., software, IT platforms)?
  - Can you think of any challenges related to the social or structural context of your organization (e.g., culture, collaboration, roles and responsibilities, competing priorities, policies)?
  - Can you think of any challenges related to the economic or legal context (e.g., funding, privacy laws, data sharing agreements)?

1. **What would help facilitate the detection of emerging drug trends in your jurisdiction sooner?**

Probe:

- - Can you think of particular data sources you wish you had access to and why?
  - Can you think of any facilitators related to human skills/knowledge and resources?
  - Can you think of any facilitators related to processes/tasks (e.g. data management, data access, aberration detection algorithms, data sharing agreements)
  - Can you think of any facilitators related to technology (e.g., access to supporting systems)?
  - Can you think of any facilitators related to the social or structural context of your organization (e.g., culture, collaboration, roles and responsibilities)?
  - Can you think of any facilitators related to the economic or legal context (e.g., funding, privacy laws)?

1. **How and with whom are emerging trends communicated internally and externally in your jurisdiction when they first emerge?**

Probe:

1. Do you have a decision tool to guide under what circumstances, how and who to notify that you can describe?

**Ending Questions**

1. **What are your thoughts on having a Pan-Canadian Drug Early Warning System?**

Probe:

- - What benefits to you foresee at the national and local level?
  - How can a national EWS best support you, what would be most helpful?

1. **Do you have any additional thoughts regarding the detection of emerging drug drugs in your jurisdiction you would like to share?**

**Demographic Question**

Before we close, I would like to take the time and ask one demographic question.

1. **How many years have you been working as an Epidemiologist or Medical Officer of Health in the area of substance use?**
2. < 1 year
3. 1-5 years
4. 6-10 years
5. > 10 years

**Closing and Wrap Up**

Thank you again for taking the time to meet with me today. I appreciate all the time and insights you have share with me on this issue. Before we close do you have any questions for me about the interview or this research? Please feel free to reach out to me via email if you have any additional comments you would like to add or if you have any questions.
